# Supplementary material for: Current Insight into Culture-Dependent and Culture-Independent Methods in Discovering Ascomycetous Taxa
Source: J Fungi (Basel). 2021 Aug 28;7(9):703. doi: 10.3390/jof7090703 (PMC8467358; doi:10.3390/jof7090703)
Supplement: Supplementary file 1 [file jof-07-00703-s001.zip › jof-1322337-supplementary.pdf]

Supplementary 1

DNA Marker Regions

The entities that make up the mycobiota consist of different cells, each of which carries its own independent genomic DNA. Fully revealing the genome sequences of all cells would be impractical in terms of time and financial resources. Therefore, some molecular markers have been determined to easily distinguish genomes with different characters. These markers consist of DNA fragments that can represent the genome without the need for complete sequencing. Interspecies differences in DNA sequences are expected to be 98% accurate in the identification of fungal species [1].

FISH (Fluorescent in situ hybridization)

The fluorescent in situ hybridization (FISH) methos is an important method used for the detection of a fungus sample growing in its natural habitat, and is one of the first metagenomic analyses used for the study of non-culturable taxa. This technique is performed using fluorescently labelled oligonucleotide probes specific to target genes in a microbial community without the need for isolation of DNA [2]. The most important advantage of this is that the taxa can be determined in a much shorter timeframe when compared to culturing methods. This method has been shown to provide reliable results in the detection of *Candida* species from blood cultures [3, 4, 5]. At present, the FISH method is more sensitive than conventional methods in identifying multiple *Candida* species, and [6] stated that the FISH method is preferred for rapid identification of *Candida* species from blood culture positive samples.

DNA Array Hybridization

Microarray technologies have been an important research application in the last decade, and have taken their place in various diagnostic fields, including the diagnosis of fungal diseases [7]. After the isolated nucleic acid from a fungal sample is denatured, it is fixed to a nylon membrane or glass slide. Then it is hybridized with the applicable probe and evaluated autoradiographically. This has the advantage of being less costly than other PCR-based methods, has unlimited capacity to accommodate oligonucleotides on a membrane, and allows for the reuse of membranes [8]. However, microarray technology is costly due to the increased error rate and the large number of materials required, as it involves different successive steps. This limits the frequency with which it is routinely used. In addition, a disadvantage of this method is that a large number of oligonucleotide probes have to be designed depending on the sequence analysis for each experiment [9].

PCR (Polymerase chain reaction)

Polymerase Chain Reaction (PCR), was first discovered by Kary Mullis in 1985, and has reformed diagnosis and biological research with its high sensitivity and original design. Mullis was awarded the Nobel Prize for this invention. The PCR technique is based on the amplification or duplication of a specific DNA sequence. It is a series of reactions carried out to enzymatically amplify a unique region between two DNA segments. The most important advantage of the system is the ability to extract millions of copies of target DNA in a very short period. There are various types of PCR, which are described below.

MT-PCR (Multiplex tandem PCR)

MT-PCR is a technology platform developed for highly multiplexed gene expression profiling and the rapid identification of clinically important pathogens. The procedure consists of two rounds of amplification. In the first step, a multiplex PCR is performed at 10 to 15 cycles to allow enrichment of the target DNA without creating competition between amplicons [10]. This product is then used as a template for the second amplification that consists of multiple individual quantitative PCR reactions with primers nested within those used for the multiplex PCR [8].

RT PCR (Real time PCR)

Real time PCR detects the initial amount of template DNA in a specific, sensitive and reproducible manner. Therefore, it is often preferred over classical PCR, in which the amount of DNA can only be determined with the final product. When the amount of fluorescence generated in each cycle is recorded, the exponential phase at the end of the PCR, where the first significant increase is

observed, can be detected. Proliferation can be distinguished at an early stage, whereas in classical PCR, the whole process is carried out and the amount can be detected by a run in agarose gel only after the final product is taken (after 30-40 cycles). While small differences in expression, even as little as two-fold, can be detected in RT-PCR, only 10-fold differences can be exposed in conventional PCR [8].

PCR-ELISA (PCR-Enzyme-linked immunoassay)

PCR-ELISA is a three-part method which includes PCR amplification, hybridization with the complementary labeled probe, and detection of reaction products in an EIA that provides either a colorimetric or a fluorescence readout. The sensitivity of PCR-EIA to detect candidemia and aspergillosis was higher than that by ethidium bromide staining, and multiple samples can be assayed in parallel [8].

RAPD (Random Amplified Polymorphic DNA)

RAPD is a type of PCR reaction, but the segments of DNA that are amplified are random. By resolving the resulting patterns, a semiunique profile can be generated from a RAPD reaction. Differentiation between Cryptococcus gatti and Cryptococcus neoformans is possible using RAPD [8].

LAMP (Loop Mediated Isothermal Amplification)

LAMP is a powerful technique that amplifies several copies of target DNA with high specificity, efficiency, and speed under isothermal conditions (with no thermal cycler) using four specially designed primers and a DNA polymerase. With the advantages of fast amplification, simple operation and easy detection, LAMP can be operated without the need for advanced equipment and personnel [8].

The most popular methods used in the investigation of environmental microbial taxa are the following: Denaturing Gradient Gel Electrophoresis (DGGE), Temperature Gradient Gel Electrophoresis (TGGE), and Terminal Restriction Fragment Length Polymorphism (T-RFLP). These are molecular fingerprinting techniques that allow for the comparison of DNA fragments in a sample. In these methods, PCR products of different sequences and/or sizes are separated by different movements on a gel or capillary [11]. It is assumed that each band or peak represents a different microbial species, and the resulting DNA patterns directly reflect the diversity within a community. At the same time, the DNA fragments belonging to the bands or peaks of interest can be selected and analyzed, and the data obtained at varying resolutions and sequence lengths can be used for phylogenetic studies [12].

Illumina

Illumina is a genome analyzer device that was introduced and it is a technique that has the potential to double data flow. Due to its high capacity, it quickly became a workhorse for whole-genome resequencing applications, including human and model organism genomic projects. The Illumina platform utilizes a sequencing-by-synthesis approach coupled with bridge amplification on the surface of a flow cell [13].

Ion Torrent Sequencing

In 2010, Life Technologies introduced the Ion Personal Genome Machine (PGM) as a postlight sequencing technology. This system relies on the real-time detection of hydrogen ion concentrations, released as a by-product when a nucleotide is incorporated into a strand of DNA by polymerase [13]. In this system, the nucleotide sequence is converted into digital information on a semiconductor chip depending on the hydrogen ion concentration.

Pacific Biosciences

Introduced by Pacific Biosciences in 2010, this technique is a third generation sequencing method aimed at sequencing long nucleotide molecules. A nanostructure called a Zero Mode Waveguide (ZMW) is used for real-time observation of DNA polymerization. It consists of tens of thousands of subwavelengths, ten-nanometre diameter holes, fabricated by perforating a thin metal film supported by a transparent substrate [13].

Oxford Nanopore

Oxford Nanopore Technologies (ONT) licensed a core nanopore sequencing patent in 2007, and began a strand sequencing effort in 2010 [14]. To date, only MinION-based strand sequencing has been successfully employed by independent genomics laboratories. The MinION is a 90-g portable device. At its core is a flow cell bearing up to 2048 individually addressable nanopores that can be controlled in groups of 512 by an application-specific integrated circuit (ASIC). Prior to sequencing, adapters are ligated to both ends of genomic DNA or cDNA fragments. These adapters facilitate strand capture and loading of an enzyme at the 5′-end of one strand. The enzyme is required to ensure unidirectional single-nucleotide displacement along the DNA strand at a millisecond time scale. The adapters also concentrate DNA substrates at the membrane surface proximal to the nanopore, boosting the DNA capture rate by several thousand [15].

References

1. Gürsoy, N.C.; Otlu, B. Molecular diagnostic tests in microbiota investigations. *J. Biotechnol. Strateg. Health Res.* **2017**, *1*, 56–67.
2. Amann, R.I.; Ludwig, W.; Schleifer, K.H. Phylogenetic identification and in situ detection of individual microbial cells without cultivation. *Microbiol Rev.* **1995**, *59*, 143–169.
3. Reller, M.E.; Mallonee, A.B.; Kwiatkowski, N.P.; Merz, W.G. Use of peptide nucleic acid-fluorescence in situ hybridization for definitive, rapid identification of five common *Candida* species. *J. Clin. Microbiol.* **2007**, *45*, 3802–3803, doi:10.1128/jcm.01127-07.
4. Shepard, J.R.; Addison, R.M.; Alexander, B.D.; Della-Latta, P.; Gherna, M.; Haase, G.; Hall, G.; Johnson, J.K.; Merz, W.G.; Peltroche-Llacsahuanga, H.; et al. Multicenter evaluation of the *Candida albicans* / *Candida glabrata* Peptide Nucleic Acid Fluorescent In Situ Hybridization Method for Simultaneous Dual-Color Identification of *C. albicans* and *C. glabrata* Directly from Blood Culture Bottles. *J. Clin. Microbiol.* **2008**, *46*, 50–55, doi:10.1128/jcm.01385-07.
5. Farina, C.; Perin, S.; Andreoni, S.; Conte, M.; Fazii, P.; Lombardi, G.; Manso, E.; Morazzoni, C.; Sanna, S. Evaluation of the peptide nucleic acid fluorescence in situ hybridisation technology for yeast identification directly from positive blood cultures: an Italian experience. *Mycoses* **2012**, *55*, 388–392.
6. Börekçi, G.; Ersöz, G.; Otağ, F.; Özturhan, H.; Şen, S.; Söylemez, F.; Akalın, H.; Özkul, Y.; Emekdaş, G. Identification of *Candida* species from blood cultures with fluorescent in situ hybridization (FISH), polymerase chain reaction-restriction fragment length polymorphism (PCR-RFLP) and conventional culture methods. *Med. J. Trakya Univ.* **2010**, *27*, 183–191.
7. Rupp, S. Microarray Technologies in Fungal Diagnostics. *Methods Mol. Biol.* **2016**, *1508*, 385–409, doi:10.1007/978-1-4939-6515-1_22.
8. Malhotra, S.; Sharma, S.; Bhatia, N.J.K.; Kumar, P.; Bhatia, N.K.; Patil, V.; Hans, C. Recent diagnostic techniques in mycology. *J. Med. Microbiol. Diagn.* **2014**, *3*, doi:10.4172/2161-0703.1000146.
9. Jaksik, R.; Iwanaszko, M.; Rzeszowska-Wolny, J.; Kimmel, M. Microarray experiments and factors which affect their reliability. *Biol. Direct* **2015**, *10*, 1–14, doi:10.1186/s13062-015-0077-2.
10. Lau, A.; Chen, S.; Sleiman, S.; Sorrell, T. Current status and future perspectives on molecular and serological methods in diagnostic mycology. *Futur. Microbiol.* **2009**, *4*, 1185–1222, doi:10.2217/fmb.09.70.
11. Gilbride, K.; Lee, D.-Y.; Beaudette, L. Molecular techniques in wastewater: Understanding microbial communities, detecting pathogens, and real-time process control. *J. Microbiol. Methods* **2006**, *66*, 1–20, doi:10.1016/j.mimet.2006.02.016.
12. Kim, J.; Lim, J.; Lee, C. Quantitative real-time PCR approaches for microbial community studies in wastewater treatment systems: Applications and considerations. *Biotechnol. Adv.* **2013**, *31*, 1358–1373, doi:10.1016/j.biotechadv.2013.05.010.
13. Shokralla, S.; Spall, J.L.; Gibson, J.F.; Hajibabaei, M. Next-generation sequencing technologies for environmental DNA research. Molecular Ecology. 2012, 21, 1794–1805. doi: 10.1111/j.1365-294X.2012.05538.x.
14. Deamer, D.; Akeson, M.; Branton, D. Three decades of nanopore sequencing. *Nat. Biotechnol.* **2016**, *34*, 518–524, doi:10.1038/nbt.3423.
15. Jain, M.; Olsen, H.E.; Paten, B.; Akeson, M. The Oxford Nanopore MinION: delivery of nanopore sequencing to the genomics community. *Genome Biol.* **2016**, *17*, 1–11, doi:10.1186/s13059-016-1103-0.
